# Perceived Paternal Acceptance–Rejection, Self-Perception, and Peer Victimization in Preadolescents with and Without Special Educational Needs

**DOI:** 10.3390/ijerph23030367

**Published:** 2026-03-13

**Authors:** Antonios I. Christou, Zacharenia Karampini, Elias Kourkoutas, Flora Bacopoulou

**Affiliations:** 1 Department of Special Education, University of Thessaly, 38221 Volos, Greece; 2 Department of Primary Education, University of Crete, 74100 Rethymno, Greece; ptpep1086@edc.uoc.gr (Z.K.); eliaskourk@uoc.gr (E.K.); 3 Clinic for Assessment of Adolescent Learning Difficulties, Center for Adolescent Medicine and United Nations Educational, Scientific and Cultural Organization, First Department of Pediatrics, Medical School, National and Kapodistrian University of Athens, 11527 Athens, Greece; fbacopoulou@med.uoa.gr

**Keywords:** peer victimization, paternal acceptance–rejection, child mental health, family risk factors, special educational needs, preadolescents, bullying prevention, psychosocial adjustment

## Abstract

**Highlights:**

**Public health relevance—How does this work relate to a public health issue?**

- Peer victimization in preadolescence is a significant public mental health concern, particularly for children with special educational needs (SEN).
- Perceived paternal acceptance–rejection emerges as a key family-level determinant of children’s vulnerability to peer victimization.

**Public health significance—Why is this work of significance to public health?**

- The study identifies distinct paternal relational pathways to victimization for children with and without SEN, highlighting differential risk mechanisms.
- Findings extend parental acceptance–rejection theory into school-based victimization research, informing family-focused prevention models.

**Public health implications—What are the key implications for practitioners, policy makers, and researchers?**

- Prevention strategies should incorporate father-focused parenting support to reduce victimization risk, especially for children with SEN.
- Public health and educational policies should promote early family-based interventions alongside school anti-bullying programs.

**Abstract:**

Peer victimization during preadolescence constitutes a significant public mental health concern, particularly for children with special educational needs (SEN). Family relational factors, and especially paternal acceptance–rejection, may influence children’s psychosocial adjustment and vulnerability to victimization. The present study examined the associations between perceived paternal acceptance–rejection, multidimensional self-perception, and peer victimization among preadolescents with and without SEN. A total of 660 students attending the final grades of Greek primary schools (553 without SEN; 107 with formally identified SEN) completed standardized self-report measures of peer victimization, perceived paternal acceptance–rejection, and self-perception domains. Separate path analyses were conducted for each group to examine direct and indirect relational pathways. Among children without SEN, perceived paternal hostility/aggression was directly associated with peer victimization and indirectly associated through behavioral conduct problems and lower school competence. In contrast, among children with SEN, the absence of paternal warmth and perceived paternal indifference/rejection were directly associated with victimization, whereas paternal hostility was not significantly associated, and self-perception did not function as a mediator. Model fit indices indicated excellent fit in both groups. These findings suggest distinct paternal relational mechanisms underlying peer victimization depending on SEN status. Interventions aimed at preventing victimization may benefit from incorporating father-focused family components alongside school-based strategies, with particular emphasis on emotional warmth and support for children with SEN.

## 1. Introduction

Peer victimization during childhood and preadolescence constitutes a significant public mental health concern, with well-documented short- and long-term consequences for emotional well-being, academic adjustment, and social functioning [1,2,3]. Exposure to bullying and victimization has been associated with internalizing difficulties such as anxiety and depression, externalizing problems, impaired peer relationships, and an increased risk for later psychopathology [2,3,4,5]. From a public health perspective, understanding the familial and psychosocial factors that heighten children’s vulnerability to victimization is critical for the development of effective prevention and early intervention strategies that extend beyond school-based approaches.

Family relationships represent a central context for children’s psychosocial development and adjustment. In particular, Interpersonal Acceptance–Rejection Theory (IPARTheory) posits that children’s perceptions of parental warmth, hostility, neglect, or rejection have profound and enduring effects on psychological functioning across cultures [6,7,8]. While maternal influences have traditionally received greater research attention, growing evidence highlights the distinct and independent role of fathers in shaping children’s emotional regulation, social competence, and behavioral adjustment [9,10,11,12]. Perceived paternal rejection, hostility, or emotional unavailability has been linked to conduct problems, low self-esteem, social withdrawal, and difficulties in peer relationships—factors that may increase susceptibility to peer victimization [10,11,12,13].

Children with special educational needs (SEN) constitute a population at heightened risk for involvement in peer victimization, either as victims, perpetrators, or both [14,15,16,17]. Neurodevelopmental, emotional, behavioral, and learning difficulties may compromise social skills, peer integration, and adaptive coping, rendering these children particularly vulnerable within peer contexts [15,18]. Research consistently indicates that children with SEN experience higher rates of bullying and victimization compared to their typically developing peers, underscoring the need to identify protective and risk factors specific to this group [14,15,16,19]. Importantly, family relational processes—including parental acceptance, emotional support, and involvement—may operate differently for children with SEN, yet remain underexplored in victimization research.

Self-perception constitutes a key psychosocial mechanism through which parental relationships may influence children’s peer experiences. Children’s perceptions of their academic competence, social acceptance, physical appearance, and behavioral adequacy are shaped through ongoing interactions with their caregivers and are significantly associated with peer adjustment and resilience [20,21,22]. Negative paternal behaviors, such as hostility or emotional withdrawal, may undermine children’s self-perception and behavioral regulation, indirectly increasing vulnerability to victimization. However, little empirical research has examined these pathways simultaneously, particularly in studies comparing children with and without SEN.

Although both maternal and paternal influences are central to children’s development, fathers have been shown to contribute uniquely to behavioral regulation, social risk-taking, and peer engagement patterns, often through distinct interaction styles and emotional communication processes [10,11,12]. In many cultural contexts, paternal behaviors may also be differentially associated with externalizing behaviors and peer-related adjustment [13]. Therefore, examining paternal acceptance–rejection independently may clarify relational mechanisms that are not fully captured in general parental models.

The present study addresses these gaps by examining associations between perceived paternal acceptance–rejection, multidimensional self-perception, and peer victimization in preadolescents with and without SEN. Using path analytic models, the study aims to identify both direct and indirect relational pathways linking paternal behaviors to victimization outcomes and to explore whether these pathways differ according to SEN status. By integrating family relational theory with a public health perspective on peer victimization, this research seeks to inform more targeted, family-inclusive prevention and intervention strategies.

## 2. Materials and Methods

### 2.1. Participants and Study Design

The study employed a cross-sectional design and was conducted with a large sample of primary school students in Greece. Participants were recruited from 10 public primary schools in cities across the island of Crete. Schools were approached through the regional educational authority and agreed to participate voluntarily. Therefore, the sampling procedure can be considered school-based convenience sampling, as participation depended on institutional approval and parental consent. All students enrolled in the final three Grades were invited to participate. Written informed consent was obtained from parents or legal guardians, and assent was obtained from children. Inclusion criteria were: (a) enrollment in mainstream education and (b) sufficient language proficiency to complete the questionnaires. Students identified with special educational needs (SEN) were classified based on official documentation following formal assessments conducted by KEDASY (Centers for Educational and Counseling Support), the state-authorized diagnostic services in Greece. All students in the SEN group had documented diagnoses and were attending inclusion classes within mainstream primary schools. The final sample consisted of 660 students (58% boys), aged 9–11 years (M = 9.8, SD = 1.1). The participation rate was approximately 28%. Of these, 553 were typically developing children, while 107 had formally identified special educational needs (SEN), including social, emotional, behavioral, and learning difficulties, based on official diagnostic documentation. The study was approved by the Greek Pedagogical Institute following submission of a detailed research protocol. Data collection was conducted within the school setting during regular school hours. Participation was voluntary, and written informed consent was obtained from parents or legal guardians prior to children’s participation. The broader research project examined children’s perceptions of relationships with parents, teachers, and peers, as well as psychosocial characteristics and involvement in peer victimization. The present study focused specifically on perceived paternal acceptance–rejection, children’s self-perception, and peer victimization. The study employed a cross-sectional design; therefore, causal inferences cannot be drawn.

### 2.2. Measures

#### 2.2.1. Peer Victimization

Peer victimization was assessed using the Peer Experiences Questionnaire (PEQ) [23], which evaluates children’s experiences of victimization and aggressive interactions with peers. The Greek version of the instrument has been translated, culturally adapted, and psychometrically validated for use with Greek school populations [24]. Higher scores indicate greater involvement in peer victimization. Internal consistency in the present sample was acceptable to good, with Cronbach’s α = 0.87 and McDonald’s ω = 0.89. Reliability indices were comparable to those reported in previous validation studies.

#### 2.2.2. Perceived Paternal Acceptance–Rejection

Children’s perceptions of their relationship with their father were measured using the Parental Acceptance–Rejection Questionnaire—Child Version (PARQ) [25], father subscale. The instrument assesses four dimensions of perceived paternal behavior: warmth/affection, hostility/aggression, indifference/neglect, and undifferentiated rejection. The Greek version of the PARQ has demonstrated satisfactory reliability and validity and has been standardized for the Greek cultural context [26]. Higher scores on hostility, neglect, and rejection reflect greater perceived paternal rejection, whereas higher scores on warmth reflect greater perceived acceptance. Internal consistency in the present sample was acceptable to good, with Cronbach’s α = 0.82 and McDonald’s ω = 0.84. Reliability indices were comparable to those reported in previous validation studies.

#### 2.2.3. Self-Perception

Children’s self-perception was assessed using the Self-Perception Profile for Children [27] (SPPC), as adapted and standardized in Greek [28]. The instrument measures multiple domains of self-perception, including school competence, social acceptance, athletic competence, physical appearance, behavioral conduct, and global self-worth. The Greek version has been widely used in educational and psychological research and exhibits satisfactory psychometric properties [28]. Internal consistency in the present sample was acceptable to good, with Cronbach’s α = 0.90 and McDonald’s ω = 0.91. Reliability indices were comparable to those reported in previous validation studies.

### 2.3. Procedure

Data were collected in classroom settings by trained researchers following standardized administration procedures. Questionnaires were administered in group format, with instructions provided both orally and in writing. Researchers were available to clarify questions when necessary, particularly for children with SEN, in order to ensure comprehension while minimizing response bias. All data were collected anonymously, and confidentiality was strictly maintained throughout the research process.

### 2.4. Statistical Analysis

Statistical analyses were conducted using IBM SPSS Statistics Version 20 [29]. Descriptive statistics were calculated for all study variables. To examine the relational pathways between perceived paternal acceptance–rejection, self-perception dimensions, and peer victimization, path analysis was employed.

Separate path models were estimated for children with and without SEN. Dimensions of perceived paternal acceptance–rejection were specified as exogenous variables, self-perception domains as endogenous mediating variables, and peer victimization as the outcome variable. Model fit was evaluated using multiple indices commonly recommended in structural equation modeling, including the chi-square statistic (χ^2^) and its associated degrees of freedom (df), the Comparative Fit Index (CFI), the Tucker–Lewis Index (TLI), the Root Mean Square Error of Approximation (RMSEA), the Standardized Root Mean Square Residual (SRMR), and the Adjusted Goodness-of-Fit Index (AGFI). Following commonly accepted guidelines, CFI and TLI values above 0.90 indicate acceptable fit and values above 0.95 indicate excellent fit, RMSEA values below 0.06 indicate good fit, and SRMR values below 0.08 indicate acceptable fit. Statistical significance was set at *p* < 0.05.

## 3. Results

### 3.1. Descriptive Statistics

Descriptive statistics were computed for all study variables, including perceived paternal acceptance–rejection dimensions, self-perception domains, and peer victimization. Preliminary screening indicated no substantial deviations from expected distributions (see Table 1). All variables were retained for subsequent path analyses.

### 3.2. Path Analysis Models

Separate path analysis models were estimated for children without SEN and for children with SEN in order to examine potential differences in relational pathways linking perceived paternal acceptance–rejection, self-perception, and peer victimization. In both models, dimensions of perceived paternal acceptance–rejection (warmth/affection, hostility/aggression, indifference/neglect, and undifferentiated rejection) were specified as exogenous variables. Self-perception domains (school competence, peer relations, athletic competence, physical appearance, behavioral conduct, and global self-worth) were specified as endogenous mediating variables, and peer victimization was specified as the outcome variable. Model fit indices indicated excellent overall fit for both models. For children without SEN, the model demonstrated excellent fit to the data (χ^2^ = 21.84, df = 18, CFI = 0.99, TLI = 0.98, RMSEA = 0.00, SRMR = 0.024, AGFI = 0.99). For children with SEN, the model also showed satisfactory to good fit (χ^2^ = 24.67, df = 18, CFI = 0.97, TLI = 0.96, RMSEA = 0.00, SRMR = 0.036, AGFI = 0.94).

### 3.3. Model 1: Children Without Special Educational Needs

For children without SEN (N = 553), perceived paternal hostility/aggression was directly and positively associated with peer victimization. In addition, paternal hostility/aggression was significantly associated with higher levels of behavioral conduct problems, which in turn were positively related to peer victimization, indicating an indirect pathway linking paternal hostility to victimization through behavioral difficulties.

**Figure 1:**
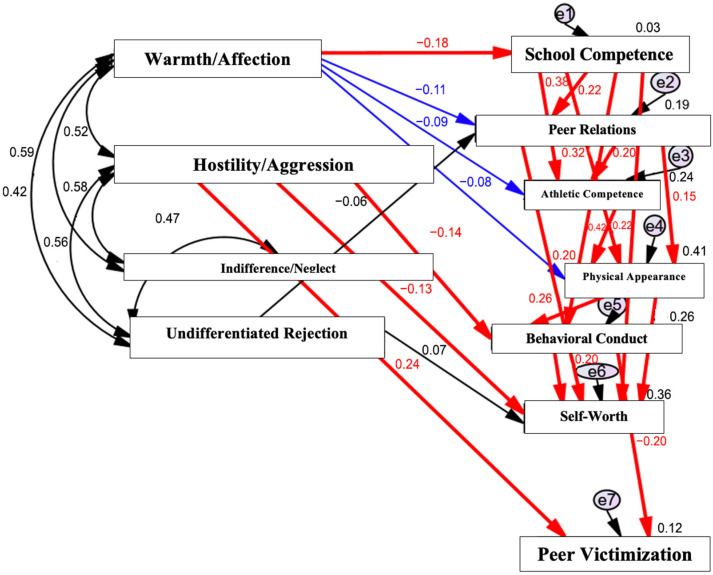
Path analysis model depicting associations between perceived paternal acceptance–rejection, self-perception domains, and peer victimization among children without special educational needs (SEN) (N = 553). Rectangles represent observed variables. Single-headed arrows indicate statistically significant direct paths, whereas double-headed arrows indicate covariances among exogenous variables. Model fit indices: RMSEA = 0.00; AGFI = 0.99. Solid lines denote statistically significant paths (*p* < 0.05), and bold lines denote highly significant paths (*p* < 0.001).

Perceived absence of paternal warmth/affection was negatively associated with children’s perceived school competence. Lower school competence was subsequently associated with higher behavioral conduct problems and increased peer victimization, indicating a multi-step indirect pathway. Furthermore, reduced school competence was associated with lower perceived physical appearance, which was also related to peer victimization. No statistically significant direct associations were observed between paternal indifference/neglect or undifferentiated rejection and peer victimization in children without SEN. The full path model for children without SEN is presented in Figure 1.

### 3.4. Model 2: Children with Special Educational Needs

**Figure 2:**
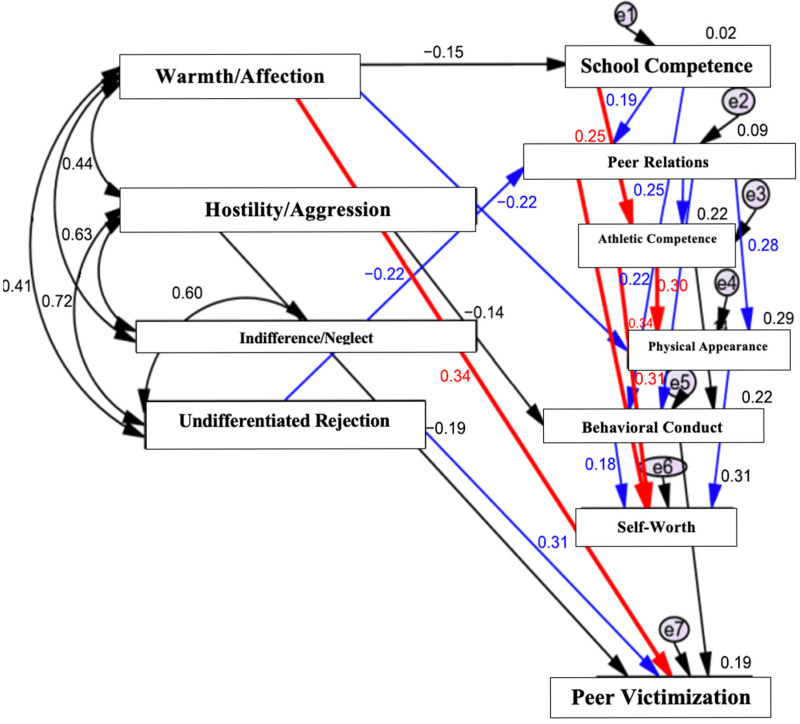
Path analysis model depicting associations between perceived paternal acceptance–rejection, self-perception domains, and peer victimization among children with special educational needs (SEN) (N = 107). Rectangles represent observed variables. Single-headed arrows indicate statistically significant direct paths, whereas double-headed arrows indicate covariances among exogenous variables. Model fit indices: RMSEA = 0.00; AGFI = 0.94. Solid lines denote statistically significant paths (*p* < 0.05), and bold lines denote highly significant paths (*p* < 0.001).

For children with SEN (N = 107), a distinct pattern of associations emerged. Perceived absence of paternal warmth/affection was directly and strongly associated with peer victimization. In addition, perceived paternal indifference/undifferentiated rejection was directly associated with higher levels of victimization. In contrast to the model for children without SEN, perceived paternal hostility/aggression and indifference/neglect were not significantly associated with self-perception domains or peer victimization in the SEN group. Moreover, self-perception domains did not function as statistically significant mediators between paternal behaviors and victimization in this group. The corresponding path model for children with SEN is shown in Figure 2.

Overall, the results indicate group-specific relational pathways linking perceived paternal acceptance–rejection to peer victimization (see also Table 2). Among children without SEN, victimization was primarily associated with perceived paternal hostility/aggression and indirect pathways involving school competence and behavioral conduct. Among children with SEN, victimization was primarily associated with the perceived absence of paternal warmth and emotional acceptance, as well as paternal indifference/rejection, with limited statistical mediation by self-perception.

## 4. Discussion

The present study examined the associations between perceived paternal acceptance–rejection, self-perception, and peer victimization among preadolescents with and without special educational needs (SEN). Using separate path analytic models for the two groups, the findings suggest that paternal relational processes are differentially associated with victimization risk depending on children’s developmental and educational profiles. Overall, the results underscore the central role of fathers in children’s psychosocial adjustment and highlight family-level mechanisms that are highly relevant to public mental health prevention efforts.

Among children without SEN, perceived paternal hostility/aggression emerged as a key factor associated with peer victimization, both directly and indirectly through behavioral conduct problems and lower school competence (Figure 1). These findings are consistent with Interpersonal Acceptance–Rejection Theory (IPARTheory), which posits that perceived parental hostility and rejection undermine children’s emotional security, self-regulation, and social functioning, thereby increasing vulnerability to maladaptive peer experiences [6,7,8]. Exposure to hostile or aggressive paternal behaviors may model coercive interaction styles, heighten emotional reactivity, and contribute to conduct problems, which in turn increase the likelihood of being targeted by peers. This interpretation is also consistent with more recent evidence indicating that family relational processes remain important correlates of bullying involvement, with parental rejection and low warmth associated with poorer psychosocial adjustment and greater victimization risk [30,31]. At the same time, contemporary research suggests that these family-level effects operate within a broader developmental ecology in which school belonging, peer integration, and classroom social dynamics may either exacerbate or buffer children’s vulnerability to victimization [32]. Thus, in children without SEN, paternal hostility may be understood not only as a direct relational risk factor, but also as part of a wider set of influences shaping behavioral adjustment and children’s position within the peer group.

The indirect pathways observed through school competence and behavioral conduct further suggest that paternal warmth plays a protective role by supporting children’s academic self-concept and behavioral regulation. Lower perceived school competence was associated with both behavioral difficulties and increased victimization, highlighting the close interconnection between academic, behavioral, and peer domains during preadolescence. These results align with prior research indicating that academic difficulties and conduct problems are salient risk factors for victimization in typically developing children [2,3,4,5].

Importantly, paternal indifference/neglect and undifferentiated rejection were not directly associated with victimization in this group, suggesting that overt hostility and aggression may be associated more saliently with peer difficulties among children without SEN. From a public health perspective, these findings suggest that addressing harsh or hostile paternal behaviors may represent a useful component of family-based prevention initiatives aimed at reducing school bullying.

In contrast, a markedly different pattern emerged for children with SEN (Figure 2). For this group, peer victimization was primarily associated with the perceived absence of paternal warmth and emotional acceptance, as well as paternal indifference/undifferentiated rejection. Paternal hostility or aggression did not show significant associations with victimization or self-perception domains. These findings suggest that, for children with SEN, emotional unavailability and the lack of supportive paternal engagement may be more consequential than overt negative behaviors.

Children with SEN often face challenges related to social competence, emotional regulation, and peer integration, which may heighten their reliance on emotionally supportive family relationships as a buffer against adverse peer experiences. The absence of paternal warmth may therefore exacerbate feelings of vulnerability, social insecurity, and isolation, increasing susceptibility to victimization. Prior research has shown that children with SEN are disproportionately exposed to bullying and victimization, and that family support can function as a critical protective factor [14,15,16,17].

Notably, self-perception domains did not operate as statistically significant mediators in the SEN group. This finding may reflect the presence of more direct pathways linking paternal emotional availability to victimization risk, or it may indicate that self-perception in children with SEN is shaped by a broader constellation of contextual influences, including school placement, peer labeling, and feedback from teachers or other professionals. In such contexts, self-evaluations may be more externally structured and less sensitive to variations in paternal behavior, thereby weakening their mediating role. Alternatively, emotional security derived from paternal warmth may exert a more immediate influence on children’s stress regulation and social vigilance processes, potentially bypassing conscious self-appraisal mechanisms.

This interpretation is consistent with recent empirical research emphasizing the protective role of supportive father–child relationships in children’s peer functioning and psychosocial adjustment. Within the last five years, studies have increasingly highlighted paternal relational quality as an important protective factor against peer victimization. For example, father–child attachment and paternal acceptance have been shown to reduce vulnerability to bullying and to buffer the impact of broader family stressors on children’s social adjustment [33,34]. In addition, recent systematic reviews of parenting correlates of bullying involvement consistently identify parental warmth and acceptance as protective factors, whereas rejection, hostility, and emotional withdrawal increase the likelihood of victimization experiences [35]. Taken together, these findings support the conceptualization of paternal acceptance–rejection as an upstream relational process that may shape children’s vulnerability to peer difficulties, particularly for children with SEN whose socio-emotional adjustment may already be influenced by multiple contextual stressors.

Although the present findings are consistent with research linking parental acceptance to children’s social adjustment [36,37,38,39], the literature is not entirely uniform. Meta-analytic evidence suggests that the strength of associations between parental warmth and peer outcomes varies depending on child characteristics and contextual factors [38]. Moreover, studies grounded in ecological frameworks have emphasized that peer victimization is shaped by multiple interacting systems, including classroom climate and peer group norms [39,40]. These mixed findings suggest that paternal acceptance may operate as one contributing relational factor within a broader developmental ecology rather than as a singular determinant of peer victimization.

Alternative explanations should also be considered. Longitudinal research suggests that the association between peer victimization and children’s self-perceptions may be bidirectional, as experiences of victimization can negatively shape children’s self-evaluations over time [41]. This raises the possibility that some of the associations observed in the present study may reflect reciprocal processes rather than strictly unidirectional effects. Moreover, the cross-sectional design does not allow for the disentanglement of temporal ordering among parenting practices, self-perceptions, and victimization experiences, and shared method variance may inflate associations when multiple constructs are assessed at a single time point [42].

Importantly, the magnitude of the observed effects was modest, suggesting that paternal acceptance and children’s self-perceptions account for part—but not all—of the variance in peer victimization. This pattern aligns with multidimensional models of social adjustment, which emphasize the combined influence of family relationships, peer dynamics, and individual cognitive and emotional processes in shaping children’s peer experiences [43].

Recent longitudinal and mechanistic research further supports the possibility of transactional pathways linking family relationships and victimization. For instance, evidence indicates that peer victimization may precede increases in rejecting parenting or reductions in parental warmth, with children’s self-esteem acting as an intermediary mechanism over time. In addition, studies of bullying victims suggest that negative family climates may reduce children’s willingness to disclose victimization or seek support, thereby amplifying the psychosocial impact of bullying independent of its direct effects on global self-worth. Taken together, these findings suggest that the associations observed in the present study may reflect reciprocal and dynamically evolving processes between family relationships, self-perceptions, and peer experiences rather than a strictly linear developmental pathway [44,45].

### 4.1. Implications for Public Health and Prevention

The present findings have important implications for public health approaches to bullying prevention and child mental health promotion, particularly by highlighting paternal relational processes as modifiable family-level targets. The differential pathways identified between children with and without SEN suggest that universal prevention strategies may be insufficient unless complemented by developmentally and neurocognitively informed family interventions.

From a clinical and educational perspective, the present results support the integration of father-focused components into family-based prevention programs. Interventions that promote paternal warmth, emotional availability, and sensitive responding may indirectly influence children’s attentional and emotional processing systems, thereby reducing risk for maladaptive peer interactions. This approach appears particularly relevant for children with SEN, for whom the absence of paternal warmth emerged as a primary association of victimization. Such interventions may include father-inclusive parenting programs that emphasize emotional attunement, positive behavioral support, and constructive conflict management. Structured parent-training models, family systems interventions, and school–family partnership programs may offer opportunities to implement preventive strategies while simultaneously evaluating their effectiveness through longitudinal or multi-method research designs.

### 4.2. Limitations and Future Directions

Several limitations of the present study should be acknowledged when interpreting the findings. First, the cross-sectional design precludes causal inferences regarding the directionality of associations between perceived paternal acceptance–rejection, self-perception, and peer victimization. Bidirectional influences are likely, whereby children’s behavioral characteristics and victimization experiences may also shape parental responses. Longitudinal designs are therefore needed to examine developmental trajectories and transactional processes over time.

Second, the study relied exclusively on child self-report measures, which may be subject to perceptual biases, shared method variance, and social desirability effects. Although children’s perceptions are central to Interpersonal Acceptance–Rejection Theory, future research would benefit from multi-informant approaches incorporating paternal reports, teacher assessments, and observational measures of parent–child interaction.

Third, the group of children with SEN was heterogeneous, encompassing a range of social, emotional, behavioral, and learning difficulties. While this reflects the ecological reality of school-based populations, it may have obscured disorder-specific pathways. Future studies should examine more diagnostically homogeneous subgroups to clarify whether distinct paternal relational mechanisms operate across different neurodevelopmental and emotional profiles.

In addition, the study focused exclusively on paternal acceptance–rejection, without parallel examination of maternal behaviors or broader family dynamics. Future research should adopt integrative family-system approaches that consider maternal acceptance–rejection, co-parenting quality, and family stressors, as well as potential moderating effects of child sex, school context, and cultural norms regarding father involvement.

Furthermore, the study was conducted within the Greek cultural and educational context, where paternal roles, family dynamics, and school systems may differ from those in other countries. Cultural norms regarding father involvement and emotional expression may influence how paternal acceptance–rejection is perceived and experienced by children. Therefore, caution is warranted when generalizing these findings to other sociocultural contexts.

Another limitation concerns the relatively modest participation rate (approximately 28%) and the sampling strategy employed in the study. Participants were recruited from schools that agreed to participate following approval from educational authorities; therefore, the sample can be considered a school-based convenience sample rather than a fully representative population sample. It is possible that families who consented to participate were more engaged with the school context or more interested in psychological research, which may introduce a degree of self-selection bias. Consequently, the sample may not fully represent the broader population of Greek primary school students, and the findings should be interpreted with caution when considering their generalizability to other school contexts.

Future work would benefit from SEN-stratified longitudinal designs with multi-informant measurement (child, parent, teacher/peer reports) to test whether paternal acceptance–rejection has differential effects across SEN subtypes (e.g., learning vs. communication vs. developmental profiles) and school contexts. Given recent evidence that social integration/classroom cohesion and community norms moderate disability-related bullying risk, modelling cross-level interactions (family × classroom × community) may clarify why some SEN pupils are disproportionately victimised. Finally, the SEND intervention literature underscores the need for co-developed, rigorously evaluated school programmes that integrate parent components and explicitly address peer inclusion and belonging [46,47].

## 5. Conclusions

The present study suggests a possible pathway where perceived paternal acceptance–rejection is differentially associated with peer victimization in children with and without special educational needs (SEN). Among children without SEN, paternal hostility and aggression were found to be associated more strongly with victimization, operating both directly and indirectly through behavioral and academic pathways. In contrast, for children with SEN, the absence of paternal warmth and emotional acceptance constituted the primary risk factor for victimization, underscoring the centrality of emotional availability in buffering social vulnerability within this group.

These findings contribute to the growing body of evidence highlighting fathers’ unique and independent role in children’s psychosocial adjustment and extend existing bullying research by identifying distinct family-related risk mechanisms across developmental profiles. From a public health perspective, the results support the need for father-focused, family-inclusive prevention strategies that complement school-based anti-bullying programs. Interventions aimed at reducing harsh paternal behaviors and strengthening paternal warmth and engagement may enhance children’s emotional regulation, social adjustment, and resilience to peer victimization. Integrating family relational dynamics into public health and educational policies may therefore represent a critical step toward more effective and inclusive approaches to bullying prevention and child mental health promotion.

## Figures and Tables

**Table 1:** Descriptive statistics (means and standard deviations) for perceived paternal acceptance–rejection, self-perception domains, and peer victimization in children with and without special educational needs (SEN).

Variable	Children Without SEN (N = 553)		Children with SEN (N = 107)	
	M	SD	M	SD
Perceived Paternal Acceptance–Rejection				
Warmth/Affection	3.62	0.64	3.21	0.71
Hostility/Aggression	1.84	0.72	2.05	0.78
Indifference/Neglect	1.67	0.69	2.32	0.81
Undifferentiated Rejection	1.59	0.65	2.18	0.77
Self-Perception Domains				
School Competence	3.31	0.58	2.71	0.63
Peer Relations	3.28	0.61	2.74	0.68
Athletic Competence	3.12	0.66	2.79	0.69
Physical Appearance	3.35	0.59	3.02	0.62
Behavioral Conduct	3.26	0.57	2.63	0.65
Global Self-Worth	3.42	0.56	2.91	0.61
Peer Victimization				
Victimization	1.91	0.73	2.56	0.84

Note. M = mean; SD = standard deviation; SEN = special educational needs.

**Table 2:** Standardized Path Coefficients for the Path Analysis Models.

Path	β	SE	*p*
Children without SEN (N = 553)			
Paternal hostility/aggression → Peer victimization	0.22	0.04	<0.001
Paternal hostility/aggression → Behavioral conduct	0.31	0.05	<0.001
Behavioral conduct → Peer victimization	0.18	0.05	0.002
Paternal warmth/affection → School competence	0.27	0.04	<0.001
School competence → Behavioral conduct	−0.24	0.04	<0.001
School competence → Physical appearance	0.29	0.04	<0.001
Physical appearance → Peer victimization	−0.14	0.05	0.011
Children with SEN (N = 107)			
Paternal warmth/affection → Peer victimization	−0.32	0.09	<0.001
Paternal indifference/neglect → Peer victimization	0.28	0.10	0.004

Note. β = standardized path coefficient; SE = standard error; SEN = special educational needs. Only statistically significant paths are presented for clarity.

## Data Availability

Data are available from the corresponding author upon reasonable request.

## Abbreviations

The following abbreviations are used in this manuscript:

SEN	Special Educational Needs
IPARTheory	Interpersonal Acceptance–Rejection Theory
PARQ	Parental Acceptance–Rejection Questionnaire
PEQ	Peer Experiences Questionnaire
SPPC	Self-Perception Profile for Children
